# Impact of High-Power and Very High-Power Short-Duration Radiofrequency Ablation on Procedure Characteristics and First-Pass Isolation During Pulmonary Vein Isolation

**DOI:** 10.3389/fcvm.2022.935705

**Published:** 2022-07-07

**Authors:** Zoltán Salló, Péter Perge, Bernadett Balogi, Gábor Orbán, Katalin Piros, Szilvia Herczeg, Klaudia Vivien Nagy, István Osztheimer, Pál Ábrahám, Béla Merkely, László Gellér, Nándor Szegedi

**Affiliations:** Heart and Vascular Centre, Semmelweis University, Budapest, Hungary

**Keywords:** high-power short-duration, very high-power short-duration, ablation, pulmonary vein isolation, atrial fibrillation, first-pass isolation

## Abstract

**Introduction:**

High-power short-duration (HPSD) radiofrequency ablation has been proposed to produce rapid and effective lesions for pulmonary vein isolation (PVI). We aimed to evaluate the procedural characteristics and the first-pass isolation (FPI) rate of HPSD and very high-power short-duration (vHPSD) ablation compared to the low-power long-duration (LPLD) ablation technique.

**Methods:**

One hundred fifty-six patients with atrial fibrillation (AF) were enrolled and assigned to LPLD, HPSD, or vHPSD PVI. The energy setting was 30, 50, and 90 W in the LPLD, HPSD, and vHPSD groups, respectively. In the vHPSD group, 90 W/4 s energy delivery was used in the QMODE+ setting. In the other groups, ablation index-guided applications were delivered with 30 W (LPLD) or 50 W (HPSD).

**Results:**

Bilateral PVI was achieved in all cases. Compared to the LPLD group, the HPSD and vHPSD groups had shorter procedure time [85 (75–101) min, 79 (65–91) min, and 70 (53–83) min], left atrial dwelling time [61 (55–70) min, 53 (41–56) min, and 45 (34–52) min], total RF time [1,567 (1,366–1,761) s, 1,398 (1,021–1,711) s, and 336 (247–386) s], but higher bilateral FPI rate (57, 78, and 80%) (all *p*-values < 0.01). The use of HPSD (OR = 2.72, 95% CI 1.15–6.44, *p* = 0.023) and vHPSD (OR = 2.90, 95% CI 1.24–6.44, *p* = 0.014) ablation techniques were associated with a higher probability of bilateral FPI. The 9-month AF-recurrence rate was lower in case of HPSD and vHPSD compared to LPLD ablation (10, 8, and 36%, *p* = 0.0001). Moreover, the presence of FPI was associated with a lower AF-recurrence rate at 9-month (OR = 0.09, 95% CI 0.04–0.24, *p* = 0.0001).

**Conclusion:**

Our prospective, observational cohort study showed that both HPSD and vHPSD RF ablation shortens procedure and RF time and results in a higher rate of FPI compared to LPLD ablation. Moreover, the use of HPSD and vHPSD ablation increased the acute and mid-term success rate. No safety concerns were raised for HPSD or vHPSD ablation in our study.

## Introduction

Since Haïssaguerre et al. (1) discovered that triggers of atrial fibrillation (AF) originate from the pulmonary veins (PVs), the non-pharmacological treatment for AF has evolved dramatically. Pulmonary vein isolation (PVI) became the backbone of the invasive treatment of AF (2, 3). Creating contiguous and durable ablation lesion set is essential to achieve favorable long-term results (3). Durable isolation of the PVs remains challenging, however, (4–6) adopting new techniques and technologies help us to achieve better results year by year (7). According to previous studies, ablation index (AI) is a valuable marker to reach a durable PVI, minimizing AF recurrence after ablation (8). AI-guided ablation enables higher energy settings by providing reliable feedback to the operator on lesion creation. The use of high-power (HP) radiofrequency (RF) applications can reduce procedural time while maintaining efficacy and safety (9–12). After creating the circumferential ablation line around the PVs with a contiguous lesion set, the presence or absence of first-pass isolation (FPI) has been evaluated in multiple studies. The presence of FPI at least one PV side was associated with a better ablation outcome, possibly due to the better durability of PVI (13–15).

We aimed to evaluate the procedural characteristics and the FPI rate of high-power and very high-power short-duration (HPSD and vHPSD) compared to low-power long-duration (LPLD) ablation techniques.

## Methods

### Patient Population

In this single-center, prospective, observational cohort study, 156 patients with symptomatic drug-refractory AF were enrolled and underwent initial PVI between January 2019 and June 2021. First, consecutive ablations were performed with the LPLD method, then patients were ablated consecutively with the HPSD, then with the vHPSD ablation settings, after the QDot Micro™ (Biosense Webster, Diamond Bar, United States) ablation catheter became available at our center. We use multiple electroanatomical mapping systems in our Institute; therefore, only PVIs performed with CARTO^®^ 3 (Biosense Webster, Diamond Bar, United States) systems were included in the study population. Patients with long-standing persistent AF or previous AF ablation were also excluded from this study.

All patients agreed to the pre-procedural imaging and the ablation procedure. For the data retrieval and analysis, the study protocol was approved by the Semmelweis University Regional and Institutional Committee of Science and Research Ethics (No.: 134/2020) and was in accordance with the Declarations of Helsinki.

### Ablation Procedure

#### Preprocedural Considerations

Indications for AF ablation were in accordance with the current guidelines (16, 17). Vitamin-K antagonists were not interrupted before the procedure; the target INR for the day of the ablation was between 2.0 and 2.5. In patients taking direct oral anticoagulants, a single dose was withheld on the morning of the procedure, and administration was resumed 4 h after the ablation unless major bleeding events occurred. All patients underwent transesophageal echocardiography (TEE) or computed tomography (CT) angiography within 48 h of the ablation procedure to exclude left atrial appendage thrombus.

#### Intraprocedural Management

**FIGURE 1:**
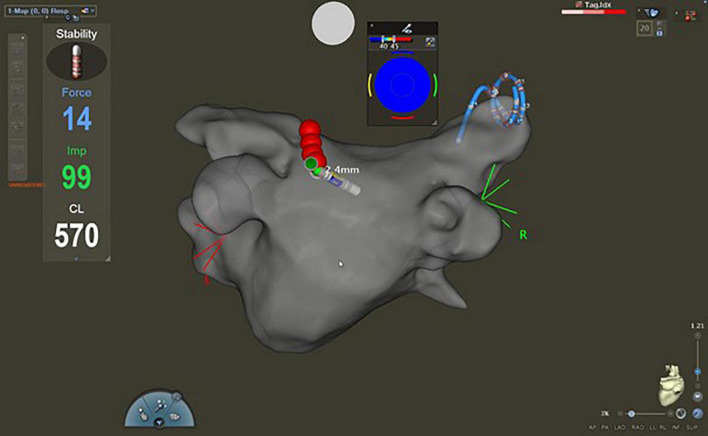
Fast anatomical map of the left atrium with CARTO^®^ 3 system (postero-anterior view). During the ablation, the Lasso™ catheter was placed in the contralateral pulmonary veins to blind the operator to the presence or absence of first-pass isolation.

The catheter ablation was carried out under local anesthesia and conscious sedation with midazolam, propofol, and fentanyl. Procedures were performed by experienced investigators (>100 PVI/year). Vascular access was obtained via the right femoral vein. A 6 Fr decapolar, steerable catheter (Inquiry™, St. Jude Medical, United States or Dynamic XT™, Boston Scientific, United States) was inserted into the coronary sinus. Subsequently, the transseptal puncture was performed using a Swartz™ SL0™ sheath (Abbott, United States) and a BRK XS transseptal needle (Abbott, United States) with fluoroscopy and pressure guidance and/or intracardiac echocardiography (ACUSON AcuNav™ Ultrasound Catheter Johnson and Johnson, United States). A second steerable sheath (Agilis NxT, Abbott, United States) was introduced via the first transseptal puncture site into the left atrium. After transseptal puncture heparin boluses were administered targeting an activated clotting time of > 300 s. Three-dimensional electroanatomic left atrial reconstruction (CARTO 3^®^, Biosense Webster, Diamond Bar, United States) was performed via fast anatomical mapping (FAM) with a multi-electrode mapping catheter (Lasso™, Biosense Webster, Diamond Bar, United States). Then, point-by-point RF ablation of each ipsilateral pair of the PVs was performed. During the RF ablation, the Lasso™ catheter was placed in the contralateral PVs to blind the operator to the presence or absence of FPI (Figure 1).

#### The Low-Power Long-Duration and High-Power Short-Duration Ablation Groups

In these patients, conventional AI-guided ablation was used. Ablation was performed in power-controlled mode with an open-irrigated tip catheter (ThermoCool SmartTouch^®^, Biosense Webster, Diamond Bar, United States). All PVIs were performed according to the CLOSE protocol (18): interlesion distance (ILD) of <6.0 mm was used (19). The target contact force was between 10 and 20 g, and the target AI was 500 on the anterior and 400 on the posterior LA wall. The VisiTag™ settings were as follows: for location stability, the minimum time was 3 s, and the maximum range was 2.5 mm; force over time was 30% with a minimum force of 3 g. The radius of the lesion tags was 3.0 mm. The RF power was set to 30 W in the LPLD group, and 50 W in the HPSD group (20). No power adjustment was made according to the LA walls. The irrigation pump setting was 30 mL/min in the HPSD group and 17 mL/min in the LPLD group.

#### The Very High-Power Short-Duration Ablation Group

In the vHPSD ablation group, the QDot Micro™ (Biosense Webster, Diamond Bar, United States) open-irrigated tip catheter was utilized. All RF applications were performed in QMODE+ mode (90 W, 4 s), in a temperature-controlled manner (21). An ILD of <5.0 mm, and a target contact force of 10–20 g was used as described previously.

#### Evaluation After Ablation

**FIGURE 2:**
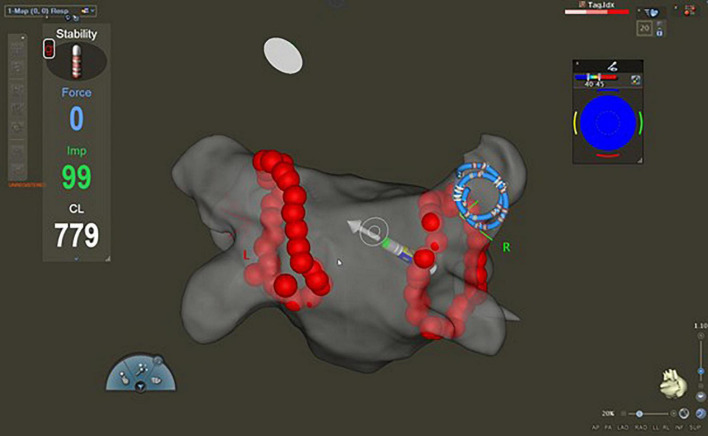
Fast anatomical map of the left atrium with CARTO^®^ 3 system (postero-anterior view). After the completion of the circumferential pulmonary vein isolation, the Lasso™ catheter was introduced into the ablated pulmonary veins to assess first-pass isolation. The Lasso™ catheter is in the right superior pulmonary vein.

After the completion of the circumferential PVI, the Lasso™ catheter was introduced into the ablated PVs to assess FPI (Figure 2). Entrance block was confirmed by the absence of sharp local PV potentials inside the PV, while the PV to left atrium conduction block was confirmed by pacing at 20 mA output with 2.0 ms pulse width from the ablation catheter placed at multiple positions in the ostia of the PV. FPI was defined as the presence of both entrance and exit block after completion of the first-pass circumferential ablation lesion set. When a residual conduction gap was found, RF applications were added to complete the PVI. The latter case was defined as FPI absent. The endpoint of the procedure was PVI as determined by entrance and exit block in all PVs after a 20-min waiting period. Adenosine was not administered (22).

#### Postprocedural Care

The patients were discharged on the day after the procedure after the evaluation of the vascular puncture site, vital functions, and echocardiography. Antiarrhythmic drugs were continued after the procedure for at least 3 months. Long-term anticoagulation therapy was managed according to the Guidelines of the European Society of Cardiology (16, 17). All patients were treated with proton-pump inhibitors for 4 weeks after ablation.

#### Follow-Up

The patient follow-ups were carried out by regular visits to our outpatient clinic at 3, 6, 9, and thereafter every 6 months, including general assessment and ECGs (12 lead, 24 h Holter).

### Statistical Analysis

The majority of the variables showed non-parametric distribution after performing the Shapiro-Wilk test. The continuous variables are presented as median with interquartile range [first quartile (Q1), third quartile (Q3)], while the categorical variables are presented as percentages with event numbers. Continuous variables were compared with the Mann-Whitney or Kruskal-Wallis tests, while categorical variables were compared with the Chi-square or Fisher’s exact test. Univariate logistic regression analysis was performed to determine the predictive value of HPSD and vHPSD ablation on the presence of FPI and success rate. Statistical analyses were performed using IBM SPSS 25 (Apache Software Foundation, United States) and GraphPad Prism 8 (GraphPad Softwares Inc., United States), software products. A two-tailed *p*-value of < 0.05 was considered statistically significant.

## Results

Overall, 156 patients were enrolled in the analysis. Table 1 summarizes the baseline characteristics. The majority of the patients had paroxysmal AF (*n* = 97, 62%); the rest presented with the persistent form. Baseline patient characteristics were balanced among the two groups. Prior stroke or transient ischemic attack was more frequent in the LPLD group, and there was a significant difference in the CHA_2_DS_2_-VASc score between the HPSD and vHPSD ablation groups.

**TABLE 1:** Baseline characteristics of the study patients.

Variable	LPLD (*n* = 53)	HPSD (*n* = 50)	vHPSD (*n* = 53)	*P*-value
Age (years)	60 (40–75)	58 (35–79)	68 (57–75)	0.3840
Sex (male)	32 (60%)	27 (54%)	33 (62%)	0.6732
Paroxysmal AF	29 (55%)	37 (74%)	31 (59%)	0.1037
Prior stroke or TIA	8 (15%)	0 (0%)	1 (2%)	**0.0015**
Diabetes mellitus	8 (15%)	7 (14%)	8 (15%)	0.9839
Hypertension	33 (62%)	37 (74%)	35 (66%)	0.4340
Congestive heart failure	2 (4%)	6 (12%)	2 (4%)	0.1472
CHA_2_DS_2_-VASc score	2 (1–3)	2 (1–3)	3 (2–4)	**0.0242**
LVEF (%)	60 (55–63)	55 (53–61)	56 (54–62)	0.1583
LA diameter (mm)	44 (39–48)	43 (38–46)	43 (41–46)	0.6935

*AF, atrial fibrillation; LA, left atrium; LPLD, low-power long duration; LVEF, left ventricular ejection fraction; HPSD, high-power short duration; TIA, transient ischemic attack. The continuous variables were expressed as medians and interquartile ranges, while the categorical variables were expressed as percentages with event numbers. The bold variables are statistically significant.*

### Procedural Characteristics

**FIGURE 3:**
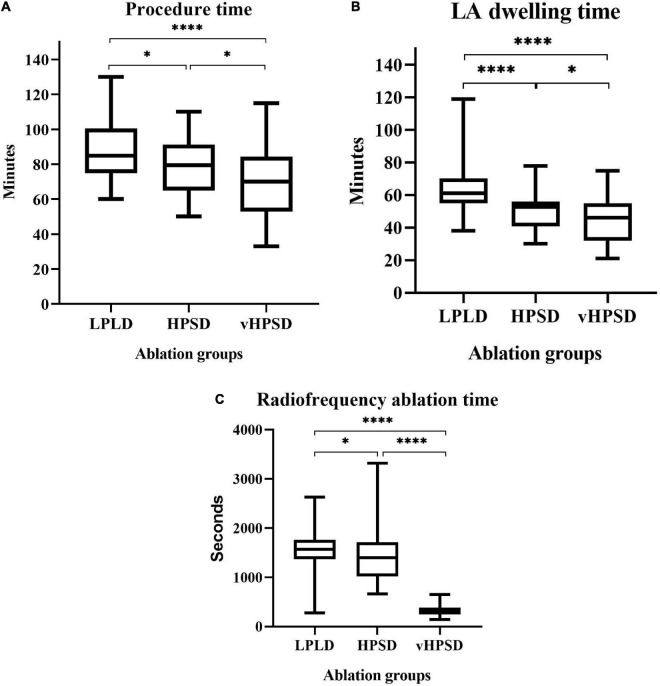
Procedure time **(A)**, left atrial dwelling time **(B)**, and radiofrequency ablation time **(C)** in the different ablation groups. Abbreviations: LDLD, low-power long-duration; HPSD, high-power short-duration; vHPSD, very high-power short-duration; LA, left atrial. *p < 0.05, ^****^p < 0.0001, ^ns^*p* ≥ 0.05.

**FIGURE 4:**
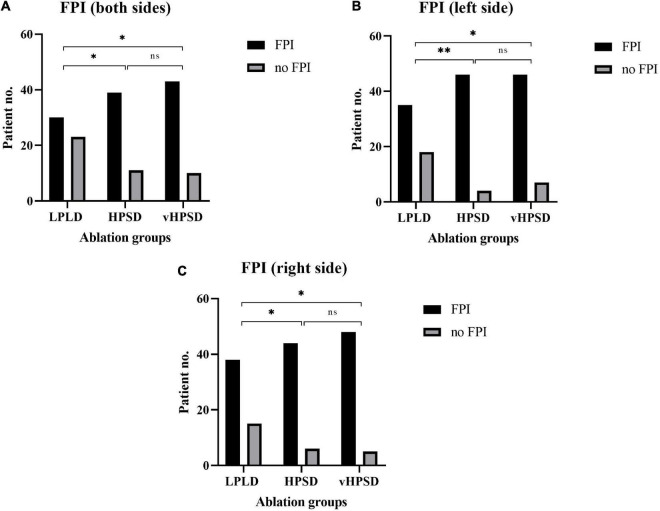
First pass isolation results in the different ablation groups (**A:** both sides; **B:** left side; **C:** right side). FPI, first-pass isolation; LDLD, low-power long-duration; HPSD, high-power short-duration; vHPSD, very high-power short-duration. *p < 0.05, ^**^p < 0.01, ^ns^*p* ≥ 0.05.

Bilateral PVI was achieved in all cases. Procedural characteristics are shown in Table 2. The procedure time was 85 (75–101) min, 79 (65–91) min, and 70 (53–83) min in the LPLD, HPSD, and vHPSD groups, respectively (*p* < 0.0001). The LA dwelling times were also decreased significantly with the increase of RF energy [61 (55–70) min, 53 (41–56) min, and 45 (34–52) min, in the LPLD, HPSD, and vHPSD groups, respectively, *p* < 0.0001]. The total RF ablation time was 1,398 (1,021–1,711) s, 1,567 (1,366–1,761) s, and 336 (247–386) s in the LPLD, HPSD, and vHPSD groups, respectively (*p* < 0.0001) (Figure 3). The total RF energy that was delivered during the procedures was 47,010 (40,980–52,830) Joules, 69,900 (51,050–85,538) Joules, and 30,240 (22,095–34,875) Joules in the LPLD, HPSD, and vHPSD ablation groups, respectively (<0.0001). The number of the RF applications were 61 (52–69), 56 (44–65), and 85 (63–99) in the LPLD, HPSD, and vHPSD ablation groups, respectively, (<0.0001). Bilateral FPI was obtained in 30 (57%) patients in the LPLD, 39 (78%) patients in the HPSD, and 43 (80%) patients in the vHPSD group (*p* = 0.0097). On the left side, FPI was achieved in 35 (66%), 46 (92%), and 46 (85%) patients in the LPLD, HPSD, and vHPSD ablation groups, respectively, (*p* = 0.0015). FPI on the right side was achieved in 38 (72%), 44 (88%), and 48 (88%) patients in the LPLD, HPSD, and vHPSD ablation groups, respectively (*p* = 0.0188). Both-sided (*p* = 0.021), left-sided (*p* = 0.0015) and right-sided (*p* = 0.0401) FPI rates were significantly higher in the HPSD group compared to the LPLD group. There was no further increase in the both-sided (*p* = 0.8080), left-sided (*p* = 0.5275), and right-sided (*p* = 0.7561) sided FPI when comparing HPSD and vHPSD (Figure 4).

**TABLE 2:** Procedural characteristics.

Ablation characteristics	LPLD (*n* = 53)	HPSD (*n* = 50)	vHPSD (*n* = 53)	*P*-value
Procedure time (min)	85 (75–101)	79 (65–91)	70 (53–83)	**<0.0001**
LA dwelling time (min)	61 (55–70)	53 (41–56)	45 (34–52)	**<0.0001**
DAP (mGym^2^)	0.16 (0.11–0.25)	0.1 (0.08–0.18)	0.17 (0.11–0.27)	**0.0014**
Ablation points	61 (52–69)	56 (44–65)	85 (63–99)	**<0.0001**
RF ablation time (s)	1,567 (1,366–1,761)	1,398 (1,021–1,711)	336 (247–386)	**<0.0001**
Total RF energy (J)	47,010 (40,980–52,830)	69,900 (51,050–85,538)	30,240 (22,095–34,875)	**<0.0001**
FPI both sides	30 (57%)	39 (78%)	43 (80%)	**0.0097**
FPI of the left PVs	35 (66%)	46 (92%)	46 (85%)	**0.0015**
FPI of the right PVs	38 (72%)	44 (88%)	48 (88%)	**0.0188**

*DAP, dose area product; LA, left atrial; LPLD, low-power long-duration group; HPSD, high-power short-duration group; LA, left atrium; RF, radiofrequency; FPI, first pass isolation; PV, pulmonary vein. The continuous variables were expressed as medians and interquartile ranges, while the categorical variables were expressed as percentages with event numbers. The bold variables are statistically significant.*

Univariate analysis revealed that the use of HPSD (both sides: OR = 2.72, 95% CI 1.15–6.44, *p* = 0.023; right side: OR = 2.90, 95% CI 1.02–8.20, *p* = 0.045; left side: OR = 5.91, 95% CI 1.84–19.04, *p* = 0.003) and vHPSD (both sides: OR = 2.90, 95% CI 1.24–6.44, *p* = 0.014; right side: OR = 3.09, 95% CI 1.09–8.74, *p* = 0.045; left side: OR = 2.89, 95% CI 1.13–7.43, *p* = 0.027) ablation techniques were associated with a higher probability of FPI.

### Follow-Up Results

All patients from the study reached the 9-month follow-up period. At this time, there was a significant difference in the AF recurrence between the three groups (36, 10, and 8% in the LPLD, HPSD, and vHPSD groups, respectively, *p* = 0.0001). Based on the univariate analysis, the use of HPSD (OR = 0.19, 95% CI 0.07–0.57, *p* = 0.003) and vHPSD (OR = 0.14, 95% CI 0.04–0.46, *p* = 0.001) ablation techniques were associated with a lower probability of AF-recurrence at 9-month. According to our findings, both-sided, right- and left-sided FPI (both sides: OR = 0.09, 95% CI 0.04–0.24, *p* = 0.0001; right side: OR = 0.09, 95% CI 0.04–0.23, *p* = 0.0001; left side: OR = 0.11, 95% CI 0.04–0.27, *p* = 0.0001) was associated with a lower probability of AF-recurrence at 9 month.

### Procedural Safety Profile

As per institutional protocol, no esophageal temperature probe was utilized during the procedures. Post-procedurally all patients received proton pump inhibitors for at least 1 month.

No steam pop occurred. We observed two groin hematomas in the LPLD group requiring no additional intervention, which resolved without sequel. There was no death, tamponade, atrio-esophageal fistula, procedural pulmonary vein stenosis/occlusion, phrenic nerve injury, stroke, thromboembolic events, myocardial infarction, or major bleeding. No complications were observed during the 9-month follow-up period.

## Discussion

### Main Results

We have shown that both HPSD and vHPSD ablation techniques are associated with a lower LA dwelling time, procedure time, and RF time than LPLD ablation. Moreover, both high-power ablation groups presented a higher FPI rate and higher 9-month AF-free survival compared to the LPLD group.

### Procedural Characteristics With High-Power Short-Duration Ablation

In the current study, the complete PVI was achieved in all patients. In addition, no complication related to RF lesion creation was observed.

In the HPSD group, both uni- and bilateral FPI rates were significantly higher than in the LPLD group; however, no significant difference was observed between the HPSD vs. vHPSD ablation settings. Furthermore, procedure time, LA dwelling time, and RF ablation time were also decreased significantly by increasing the RF power. Data are conflicting on the necessity of power adjustment on the posterior wall. We did not use any power adjustment; still, no major complications occurred (7, 20, 23).

We know from previous studies that FPI is an important predictor of long-term success rate (13–15). Vassallo et al. (24) investigated patients who underwent AF ablation with HP (50 W on the anterior wall and 45 W elsewhere in the left atrium) or LP (20 W on the posterior wall, 30 W elsewhere) RF ablation power settings. Bilateral FPI was increased significantly with HPSD ablation compared to the LPLD patients (87.32 vs. 23.29%, *p* < 0.00001), which also resulted in a higher success rate at 12 months (87.32 vs. 67.12%, *p* < 0.0039).

The PVI procedure time was also decreased significantly with HP (50 W) ablation in a study by Bunch et al. compared to conventional LP (30 W) ablation settings (104.3 ± 63.6 min vs. 170.8 ± 59.2 min; *p* < 0.0001) (25). Another study conducted by Wielandts et al. (26) randomized 96 patients to HPSD (45 W) or LPLD (35 W) ablation groups showed lower fluoroscopy dosage in the case of HPSD PVI [DAP of 1,915 (938–3,654) mGycm^2^ and 1,804 (1,225–3,176) mGycm^2^; *p* = 0.69]. In addition, this group also found that RF application time was lower in the HPSD group [16 (14–18) min vs. 26 (22–30) min; *p* < 0.001].

The vHPSD ablation technique (90 W, 4 s) was evaluated first in the QDOT-Fast clinical trial, resulting in a substantially shorter total procedure, ablation, and RF application times compared with previous studies (27). The fast and furious—AF study provided further data about the vHPSD ablation method and the QDot Micro™ (Biosense Webster, Diamond Bar, United States) ablation catheter. Twenty-eight consecutive patients underwent vHPSD PVI and were compared to 28 consecutive patients treated with conventional CF-sensing catheters utilizing the AI. All PVs were successfully isolated using vHPSD. The median RF ablation time was 338 (286–367) s vs. 1,580 (1,350–1,848) s, (*p* < 0.0001), the median procedure duration was 55 (48–60) min vs. control 105 (92–120) min, (*p* < 0.0001) in the vHPSD vs. control group, respectively. No differences in periprocedural complications were observed (21). These results are congruent with our current findings.

### Lesion Creation With High-Power Ablation

Ablation index is calculated by a weighted formula including CF, RF time, and power so that a higher power application can reach the target AI with a shorter duration. Even with high-energy RF applications, lesion creation can be properly monitored in real-time. The decrease in RF time per application makes it easier to maintain a stable catheter position, resulting in a reduced time to complete the circumferential ablation line around the PVs. Different power levels are presented in the literature for HPSD ablation, ranging from 40 to 90 W.(28). The AI was originally validated for a maximum of 45 W; however, multiple studies have been published using 50 W power and AI guidance, with great safety and efficacy profile (20).

According to our data and the literature (15, 29), ablations with higher power were more likely to be successful in the left atrium, which is in accordance with the results of previous experimental studies (30, 31). It is well-known that HPSD ablation results in a different lesion geometry (e.g., larger diameters but smaller depth) compared to conventional lower power ablation. Still, the depth of the HP applications is sufficient to create transmural lesions in the atria (31, 32). Our results comparing LPLD (30 W) and HPSD (50 W) ablation complement the findings of Hoffmann et al., who investigated the desirable target ILD during PVI (the power setting was 30 W throughout the study). Their results indicated that an ILD of 3.0–4.0 mm is associated with a better 1-year outcome compared to an ILD of 5.0–6.0 mm during CLOSE-protocol guided PVI. FPI was achieved in 35.0% in the “5–6 mm” group and 90.9% in the “3–4 mm” group (*p* < 0.0001) (19). In our present study, we used ILD < 6 mm both in LPLD and HPSD (50 W) groups, and according to our findings, HPSD ablation improves the chance of creating a contiguous lesion set, probably due to wider RF ablation lesions, which seem to increase the FPI rate by boosting the continuity of the circumferential lesions (33). Of note, lesion size might be smaller in case of vHPSD (34). For this reason, we decided to use more closely spaced ablation points in case of vHPSD (ILD < 5 mm), which resulted in a higher number of RF applications in the vHPSD group compared to the HPSD group. The legitimacy of this modification in the protocol is validated by the similar FPI rate. The overall delivered RF energy was the lowest in the vHPSD, followed by the LPLD, and the highest in the HPSD ablation group in our study. With the conventional LPLD ablation, we used higher contact force, while in cases of HPSD ablations, we aimed for lower contact force values in the target range (10–20 g) to increase the procedural safety. This may be the reason that there is no enormous difference in RF energy at 30 vs. 50 W. Of note, the target AI was the same in these two ablation groups. Limited data are available regarding the total energy delivery in case of LPLD, HPSD, and vHPSD. Nakagawa et al. reported that lesion diameter might be the largest with LPLD ablations, which was associated with an increased energy delivery (34). However, in that experimental study, the LPLD and HPSD lesions were not created with AI guidance but simply guided by the RF application duration. Thus, data on the total energy delivery cannot be transferred directly from their results to clinical AI-guided ablations.

## Limitations

This was an observational, relatively small sampled, but prospective study involving consecutive patients. Another limitation is the non-randomized nature of the study. As we did not perform redo procedures, we are not able to present data regarding potential PV reconnections. Moreover, because the efficacy of the procedures was not evaluated with continuous rhythm monitoring but with periodical Holter ECG recordings, there is a chance that some AF recurrences were not detected.

## Conclusion

Our prospective, observational cohort study showed that both HPSD and vHPSD RF ablation shortens procedure time and RF time, and results in a higher rate of FPI compared to LPLD ablation. Moreover, using HPSD and vHPSD ablation techniques increased the acute and mid-term success rates. No safety concerns were raised for HPSD or vHPSD ablation in our study.

## Data Availability Statement

The raw data supporting the conclusions of this article will be made available by the authors, without undue reservation.

## Ethics Statement

The studies involving human participants were reviewed and approved by the Semmelweis University Regional and Institutional Committee of Science and Research Ethics. The ethics committee waived the requirement of written informed consent for participation.

## Author Contributions

ZS made the basic conception, collected and analyzed the data, and wrote the manuscript. PP helped analyzing the data. BB and GO helped in data collection. KP, SH, KN, IO, and PÁ revised the article. BM revised and approved the manuscript to be published. LG and NS helped in the conception phase, made critical revision of the article, and approved the final version to be published. All authors contributed to the article and approved the submitted version.

## Conflict of Interest

ZS, NS and LG were received consultation fees from Biosense Webster. The remaining authors declare that the research was conducted in the absence of any commercial or financial relationships that could be construed as a potential conflict of interest.

## Publisher’s Note

All claims expressed in this article are solely those of the authors and do not necessarily represent those of their affiliated organizations, or those of the publisher, the editors and the reviewers. Any product that may be evaluated in this article, or claim that may be made by its manufacturer, is not guaranteed or endorsed by the publisher.
